# Exploiting powder X-ray diffraction for direct structure determination in structural biology: The P2X4 receptor trafficking motif YEQGL

**DOI:** 10.1016/j.jsb.2011.03.001

**Published:** 2011-06

**Authors:** Kotaro Fujii, Mark T. Young, Kenneth D.M. Harris

**Affiliations:** a School of Chemistry, Cardiff University, Park Place, Cardiff CF10 3AT, Wales, United Kingdom; b School of Biosciences, Cardiff University, Museum Avenue, Cardiff CF10 3AX, Wales, United Kingdom

**Keywords:** γ-Abu, γ-aminobutyric acid, AP2, clathrin adaptor protein complex 2, CD, circular dichroism spectrometry, Piv, pivaloyl, Φ, any hydrophobic amino acid, P2X, Powder X-ray diffraction, Trafficking, Direct-space structure solution, Genetic algorithm, Structure determination

## Abstract

We report the crystal structure of the 5-residue peptide acetyl-YEQGL-amide, determined directly from powder X-ray diffraction data recorded on a conventional laboratory X-ray powder diffractometer. The YEQGL motif has a known biological role, as a trafficking motif in the C-terminus of mammalian P2X4 receptors. Comparison of the crystal structure of acetyl-YEQGL-amide determined here and that of a complex formed with the μ2 subunit of the clathrin adaptor protein complex AP2 reported previously, reveals differences in conformational properties, although there are nevertheless similarities concerning aspects of the hydrogen-bonding arrangement and the hydrophobic environment of the leucine sidechain. Our results demonstrate the potential for exploiting modern powder X-ray diffraction methodology to achieve complete structure determination of materials of biological interest that do not crystallize as single crystals of suitable size and quality for single-crystal X-ray diffraction.

## 1 Introduction

Single-crystal X-ray diffraction has underpinned many major breakthroughs in structural biology (Ban et al., 2000, Crowfoot Hodgkin et al., 1955, Deisenhofer et al., 1984, Kendrew et al., 1958, Perutz et al., 1960, Schluenzen et al., 2000, Wimberly et al., 2000), although it is important to recognize that an intrinsic limitation of this technique is the requirement to prepare a single-crystal specimen of sufficient size, quality and stability. Unfortunately, for many molecules of biological interest, the preparation of suitable single crystals can represent an insurmountable challenge, and under such circumstances, structure determination by single-crystal X-ray diffraction becomes impossible. In such situations, how can progress be made to determine the structural properties of the material of interest?

In solid-state and materials sciences, the application of single-crystal X-ray diffraction is also subject to the limitation of obtaining a suitable single-crystal specimen, but in these fields there has been considerable progress in recent years in the opportunities for carrying out structure determination directly from *powder* X-ray diffraction data, circumventing the need to prepare a suitable single crystal of the material of interest. In the case of organic molecular materials, recent advances in techniques for *ab initio* structure determination from powder X-ray diffraction data (Altomare et al., 2008, Brodski et al., 2005, Brunelli et al., 2003, Chernyshev, 2001, David and Shankland, 2008, David et al., 2002, Favre-Nicolin and Černý, 2004, Hammond et al., 1997, Harris, 2003, Harris, 2009, Harris and Cheung, 2004, Harris et al., 1994, Huq and Stephens, 2003, Kariuki et al., 1996, Lightfoot et al., 1992, Tremayne, 2004, Tsue et al., 2007) are such that the structural properties of organic materials of moderate complexity can now be established relatively routinely by this approach (in particular by exploiting the direct-space strategy for structure solution), creating the opportunity to elucidate the structural properties of a wide range of materials that are unsuitable for investigation by single-crystal X-ray diffraction.

Among the range of structures that have been determined previously from powder X-ray diffraction data using the direct-space strategy, there are a number of oligopeptide structures, including Phe–Gly–Gly–Phe (Tedesco et al., 2000), Piv–^L^Pro–Gly–NHMe (Tedesco et al., 2001) and Piv–^L^Pro–γ-Abu–NHMe (Cheung et al., 2002). In addition, crystal structures of cyclic-beta-peptides have also been determined directly from powder X-ray diffraction data using traditional (reciprocal space) structure solution techniques (Seebach et al., 1997).

**Fig. 1:**
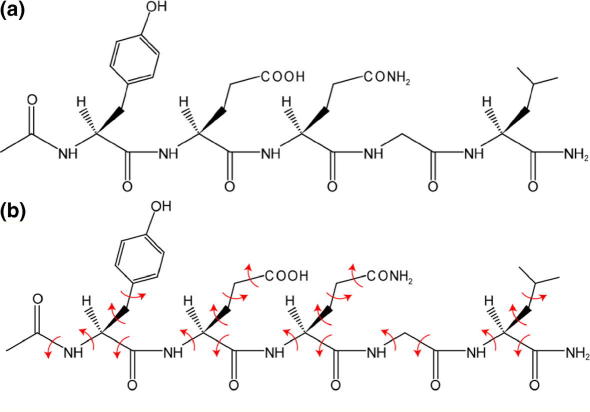
(a) The acetyl-YEQGL-amide peptide. (b) Definition of the torsion-angle variables (indicated by red arrows) in our direct-space structure-solution calculations for acetyl-YEQGL-amide from powder X-ray diffraction data (For interpretation of the references to color in this figure legend, the reader is referred to the web version of this paper.)

Our previous structural studies of peptide materials by powder X-ray diffraction were selected primarily to address specific structural issues, rather than to resolve important biological questions, and we are now focusing on applying this structure determination strategy to tackle structural problems of greater biological importance. In the present paper, we exploit modern powder X-ray diffraction methodology to achieve complete structure determination of a model oligopeptide with a known biological role, YEQGL (Fig. 1a), which is the trafficking motif in the C-terminus of mammalian P2X4 receptors.

We note that the crystal structure of YEQGL was determined here from powder X-ray diffraction data recorded on a conventional laboratory X-ray powder diffractometer, underlining the fact that it is generally feasible to carry out structure determination of organic materials using powder X-ray diffraction data recorded on a conventional laboratory instrument. In this field, it is only under certain specific circumstances that the use of data recorded at a synchrotron source becomes essential (for a more detailed discussion of this issue, see: Harris and Cheung, 2004). We also note that the crystal structure of YEQGL represents one of the most complex structural problems (with 21 torsion-angle variables required to define the molecular conformation) that has so far been reported from powder X-ray diffraction data by the direct-space structure solution technique.

### 1.1 Biological background

P2X receptors are plasma membrane ATP-gated cation channels, which play key physiological roles in nerve transmission, pain sensation and inflammation, and are important drug targets for the treatment of inflammatory and neuropathic pain (Khakh and North, 2006). A functional P2X receptor is formed from three subunits. Each subunit has intracellular N- and C-termini, two transmembrane-spanning α-helices, and a large *ecto*-domain, which contains the binding sites for ATP (Surprenant and North, 2009). The highly variable intracellular C-terminal domains of P2X receptors contain motifs that are important for downstream signaling, plasma membrane targeting and protein trafficking (Denlinger et al., 2001, Surprenant and North, 2009). The P2X4 receptor subtype undergoes constitutive recycling between the plasma membrane and endosomes (Bobanovic et al., 2002). This process is mediated by the clathrin adaptor protein complex AP2, and results in most of the receptor being found on intracellular membranes (Royle et al., 2005). The μ2 subunit of AP2 is known to recognize the canonical four amino-acid consensus sequence YXXΦ, where X is any amino acid and Φ represents a hydrophobic amino acid such as leucine (Bonifacino and Dell’Angelica, 1999). Royle et al. demonstrated that the μ2 subunit of AP2 could also recognize the non-canonical five amino-acid motif YEQGL present in the C-terminus of P2X4 (residues 378–382), and solved the crystal structure (by single-crystal X-ray diffraction) of μ2 in complex with a 10-residue peptide (VEDYEQGLSG; residues 375–384 of rat P2X4), demonstrating that the YEQGL motif could be accommodated within the binding site (Royle et al., 2005). However, the native structure of the YEQGL motif has never been determined.

### 1.2 Background to structure determination from powder X-ray diffraction data

Although single-crystal and powder X-ray diffraction patterns contain essentially the same information, in the single-crystal case this information is distributed in three-dimensional reciprocal space, whereas in the powder case the three-dimensional diffraction data are effectively compressed into one dimension (intensity *versus* diffraction angle 2*θ*) as a consequence of the random orientational distribution of the crystallites in the powder sample. Such compression of the three-dimensional diffraction data into one dimension usually results in extensive overlap of peaks in a powder X-ray diffraction pattern, particularly for structures with large unit cells and low symmetry, which is generally the case for molecular crystal structures. As a consequence of peak overlap, the process of extracting intensity data from the powder X-ray diffraction pattern can be associated with unreliability, leading to difficulties in attempting to carry out structure solution using such data.

In the present day, most reported crystal structure determination of organic molecular solids from powder X-ray diffraction data employs the direct-space strategy for structure solution (Harris et al., 1994), in which the task of structure solution is transformed into a “global optimization” problem. In the direct-space strategy (David and Shankland, 2008, Harris and Cheung, 2004), trial crystal structures are generated in direct space, independently of the experimental powder X-ray diffraction data, and the quality of each trial structure is assessed by direct comparison between the powder X-ray diffraction pattern calculated for the trial structure and the experimental powder X-ray diffraction pattern. This comparison is quantified using an appropriate figure-of-merit. In our implementations of the direct-space strategy, the figure-of-merit is the weighted powder profile *R*-factor *R*_wp_, which considers the entire digitized intensity profile point-by-point, rather than the integrated intensities of individual diffraction maxima, and thus takes peak overlap implicitly into consideration. Clearly, this approach circumvents the need to extract the intensities of individual reflections from the experimental powder X-ray diffraction pattern.

In a direct-space structure-solution calculation, each trial structure is defined by a set (***Γ***) of structural variables, which represent the position, orientation and intramolecular geometry of each molecule in the asymmetric unit. In general, the position of the molecule is defined by three coordinates {*x*, *y*, *z*} and the orientation is defined by three rotation angles {*θ*, *φ*, *ψ*}. The bond lengths and bond angles of the molecule are fixed at standard values, and the intramolecular geometry is specified by a set of variable torsion angles {*τ*_1_, *τ*_2_, ..., *τ_n_*} to define the molecular conformation. Thus, in general, for each molecule in the asymmetric unit, there are 6 + *n* variables, ***Γ*** = {*x*, *y*, *z*, *θ*, *φ*, *ψ*, *τ*_1_, *τ*_2_, ..., *τ_n_*}.

The aim of the direct-space strategy is to find the trial crystal structure that corresponds to lowest *R*-factor, and is equivalent to exploring a hypersurface *R*_wp_(***Γ***) to find the global minimum. In principle, any technique for global optimization may be used, and our current work in this field has focused on the implementation of genetic algorithm (GA) techniques for direct-space structure solution (Habershon et al., 2003, Harris et al., 1998, Harris et al., 2004, Kariuki et al., 1997, Turner et al., 2000).

The aim of *structure solution* is to obtain a good approximation to the correct crystal structure, which serves as a starting point for the subsequent *structure refinement* stage of the structure determination process, leading to a more accurate, higher quality description of the structure. The assumptions made in direct-space structure-solution calculations concerning the fixed values of bond lengths and bond angles are relaxed subsequently in the Rietveld refinement stage. For powder X-ray diffraction data, structure refinement is carried out routinely using the Rietveld refinement technique (McCusker et al., 1999, Rietveld, 1969, Young, 1993).

## 2 Materials and methods

HPLC-purified acetyl-YEQGL-amide was purchased from the University of Bristol Peptide Synthesis Facility (Bristol, UK) and supplied as lyophilized powder. The sample of acetyl-YEQGL-amide was crystallized by slow evaporation from aqueous solution, yielding very small needle-shaped crystals (width less than *ca*. 10 μm).

Powder X-ray diffraction data were recorded at ambient temperature on a Bruker D8 diffractometer operating in transmission mode (Ge-monochromated CuK_α1_ radiation; *λ *= 1.5406 Å; linear position-sensitive detector covering 12° in 2*θ*; 2*θ* range 3.5°–70°; step size 0.017°; data collection time 17 h).

Thermogravimetric analysis (TGA) and differential scanning calorimetry (DSC) were carried out on a TA Instruments Q600 Simultaneous TGA/DSC instrument for an accurately weighed sample (*ca*. 10 mg) heated at 3 °C min^−1^. A mass loss of 2.94% was observed at *ca*. 41.5 °C (corresponding to an endothermic peak in the DSC measurement), in close agreement with the calculated mass percentage of water (2.70%) in a monohydrate of acetyl-YEQGL-amide.

Circular dichroism (CD) spectroscopy was performed on an Applied Photophysics Chirascan Circular Dichroism Spectrometer using a peptide concentration of 0.64 mM in 25 mM potassium phosphate (pH 7.5) in a quartz cuvette (0.1 cm path length, Helma). CD spectra were recorded in the wavelength range from 190 nm to 400 nm, and the buffer signal was auto-subtracted.

### 2.1 Structure determination of acetyl-YEQGL-amide

**Fig. 2:**
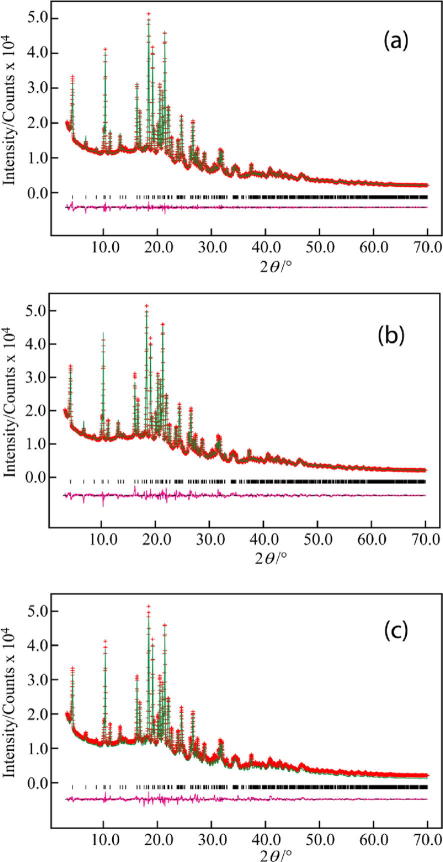
Results from powder X-ray diffraction analysis of acetyl-YEQGL-amide: (a) Le Bail refinement, (b) Rietveld refinement with the water molecule excluded from the structural model, and (c) the final Rietveld refinement with the water molecule included in the structural model. Each plot shows the experimental powder X-ray diffraction profile (red + marks), the calculated powder X-ray diffraction profile (green solid line) and the difference profile (purple, lower line). Tick marks indicate peak positions (For interpretation of the references to color in this figure legend, the reader is referred to the web version of this paper.)

The powder X-ray diffraction pattern of acetyl-YEQGL-amide was indexed using the program DICVOL04 (Boultif and Louër, 2004) (M20 = 22.1, F20 = 48.2), giving the following unit cell with monoclinic metric symmetry: *a *= 21.34 Å, *b *= 16.91 Å, *c *= 4.84 Å, *β *= 109.6° (*V *= 1645.1 Å^3^). Given the volume of this unit cell and consideration of density, the number of formula units in the unit cell was assigned as *Z *= 2. From systematic absences, the space group was assigned as *P*2_1_ (corresponding to *Z*′ = 1). Profile fitting using the Le Bail method (Le Bail et al., 1988) gave a good quality of fit (*R*_wp_ = 1.98%, *R*_p_ = 1.49%; Fig. 2a). The refined unit cell and profile parameters obtained from the Le Bail fitting procedure were used in the subsequent structure-solution calculation.

Structure solution was carried out using the direct-space genetic algorithm (GA) technique incorporated in the program EAGER (Albesa-Jové et al., 2004, Cheung et al., 2006, Fujii et al., 2010, Guo and Harris, 2005, Guo et al., 2008, Kariuki et al., 1999, Pan et al., 2006, Tedesco et al., 2003). In the GA structure-solution calculation, the acetyl-YEQGL-amide molecule was defined by a total of 26 structural variables – two positional variables (for space group *P*2_1_, the origin can be fixed arbitrarily along the *b*-axis, and thus the translational variable along the *b*-axis was fixed), three orientational variables, and 21 torsion-angle variables (Fig. 1b). The torsion angles (H–N–C=O) defining the conformations of the amide groups were fixed at 180°, corresponding to the conformation normally observed in peptide structures. Each GA structure-solution calculation involved the evolution of 100 generations for a population of 1000 structures, with 500 mating operations and 250 mutation operations carried out per generation. Eight independent GA calculations were carried out, with the same good-quality structure solution obtained in two cases.

To establish the location of the water molecule in the crystal structure, further GA calculations were carried out with a water molecule introduced into the structural model, and with the acetyl-YEQGL-amide molecule fixed at the position established from the GA calculation discussed above. However, this approach did not yield a reasonable position for the water molecule. Instead, the water molecule was located by inspection of the structure solution, based on the fact that the water molecule may be expected to be engaged in hydrogen bonding with the acetyl-YEQGL-amide molecule. On this basis, a void with sufficient space to accommodate a water molecule was identified. When located in this void, the water molecule forms hydrogen bonds with the sidechains of the tyrosine and glutamine residues of the acetyl-YEQGL-amide molecule.

This monohydrate structure of acetyl-YEQGL-amide was used as the initial structural model for Rietveld refinement, which was carried out using the GSAS program (Larson and Von Dreele, 1994). Standard restraints were applied to bond lengths and bond angles, planar restraints were applied to aromatic rings and a global isotropic displacement parameter was refined. The final Rietveld refinement gave a good fit to the powder X-ray diffraction data (*R*_wp_ = 3.15%, *R*_p_ = 2.21%, R_F_^2^ = 7.96%; Fig. 2c), with the following refined parameters: *a *= 21.3723(15) Å, *b *= 16.9359(8) Å, *c *= 4.84290(22) Å, *β *= 109.5541(35)°; *V *= 1651.84(20) Å^3^ (2*θ* range, 3.02–77.00°; 3936 profile points; 148 refined variables). For comparison, Rietveld refinement with the water molecule excluded from the structure gave a significantly inferior fit (*R*_wp_ = 3.67%, *R*_p_ = 2.71%, R_F_^2^ = 9.88%; Fig. 2b), supporting the assignment that the correct position of the water molecule had been found.

## 3 Results and discussion

### 3.1 Structural properties of acetyl-YEQGL-amide

**Fig. 3:**
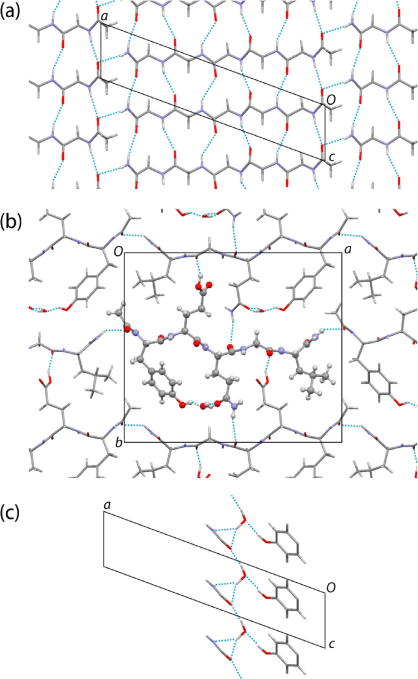
Crystal structure of acetyl-YEQGL-amide viewed (a) along the *b*-axis, showing only the backbone of the peptide, (b) along the *c*-axis, and (c) along the *b*-axis, showing only the residues engaged in hydrogen bonding with the water molecule. Hydrogen bonds are indicated by blue dotted lines (For interpretation of the references to color in this figure legend, the reader is referred to the web version of this paper.)

In the crystal structure determined here from powder X-ray diffraction data (Fig. 3), the acetyl-YEQGL-amide molecule adopts a linear conformation aligned essentially along the *a*-axis. Along the short axis of the unit cell (the *c*-axis), adjacent molecules are related by translation and give rise to a parallel β-sheet structure along this axis, involving six intermolecular N–H^…^O hydrogen bonds between each pair of adjacent molecules (N^…^O distances in the range 2.90–3.18 Å; Fig. 3a). Along the *a*-axis, each end of the molecule is connected to the neighbouring molecule by an N–H^...^O hydrogen bond involving the C=O group of the acetyl terminus and the NH_2_ group of the amide terminus (N^...^O distance, 2.92 Å). These N–H^...^O hydrogen bonds, together with the parallel β-sheet structure along the *c*-axis, give rise to a two-dimensional hydrogen-bonded sheet parallel to the *ac*-plane (Fig. 3a). Adjacent sheets of this type are bridged by two hydrogen bonds that link neighbouring molecules along the *b*-axis (Fig. 3b), specifically: (i) an O–H^...^O interaction involving O–H of glutamic acid as the donor and O=C of glycine as the acceptor (O^...^O distance, 2.65 Å), and (ii) a (rather long) N–H^...^O interaction involving an N–H bond of the sidechain of the glutamine residue as the donor and O=C of the glutamine residue on the backbone of the neighbouring molecule as the acceptor (N^...^O distance, 3.31 Å).

The water molecule effectively links the tyrosine and glutamine residues within a given molecule, and is engaged in hydrogen bonding with the OH group of tyrosine and the CONH_2_ group of glutamine (Fig. 3c). Thus, the water molecule acts as an acceptor in an O–H^...^O hydrogen bond involving tyrosine as the O–H donor (O^...^O distance, 2.72 Å), and one OH bond of the water molecule is the donor in a bifurcated hydrogen-bonding arrangement involving both the O and N atoms of the CONH_2_ group of glutamine in the same molecule as the acceptors (O–H^...^O hydrogen bond: O^...^O distance, 2.83 Å, O–H^...^O angle, 150.1°; O–H^...^N hydrogen bond: O^...^N distance, 3.05 Å, O–H^...^N angle, 144.3°). The other O–H bond of the water molecule is the donor in an O–H^...^O hydrogen bond (O^...^O distance, 2.71 Å) to the oxygen atom of the CONH_2_ group of glutamine in the adjacent molecule within the β-sheet (i.e. along the *c*-axis). The *i*-propyl side-chain of the leucine residue occupies a local hydrophobic region of the structure (see Fig. 3b), bounded by the CH_2_ group of the tyrosine residue of one neighbouring molecule, and the CH_2_ group of the glutamic acid residue and the CH_3_ group of the acetyl terminus of another neighbouring molecule.

### 3.2 Structural comparison between acetyl-YEQGL-amide and the YEQGL-μ2 complex

**Fig. 4:**
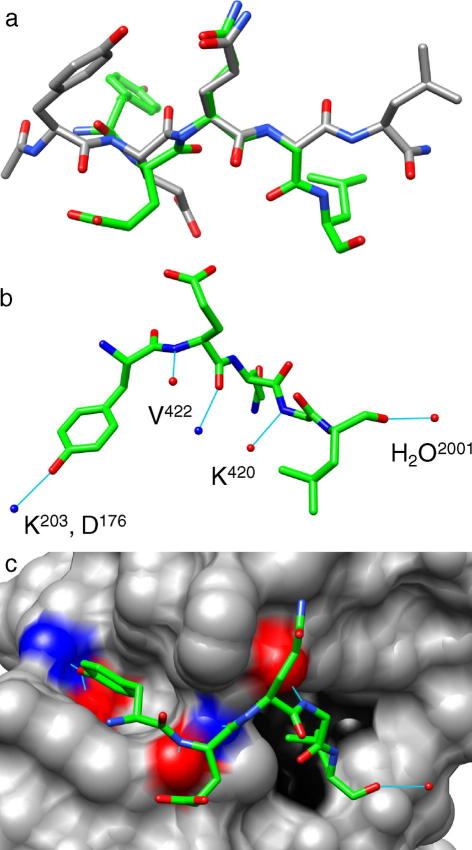
(a) Wire-frame comparisons of the crystal structure of acetyl-YEQGL-amide (gray) and the crystal structure of YEQGL in complex with the μ2 subunit of adaptor protein 2 (green; Royle et al., 2005; PDB code 2BP5). The sidechain and backbone of glutamine and the backbone of glycine are well aligned with each other, but there is considerable divergence in the positions of tyrosine, glutamic acid and leucine. (b) Wire-frame representation of the YEQGL peptide, with hydrogen bonding to the μ2 protein shown in thin light-blue lines and indicating the residues of the μ2 protein involved in hydrogen bonding. (c) Wire-frame representation of YEQGL in the binding site of the μ2 protein (gray surface representation). Atoms engaged in hydrogen bonding with YEQGL are colored red (oxygen) and blue (nitrogen). The hydrophobic pocket responsible for binding leucine is shown in black. (For interpretation of the references to color in this figure legend, the reader is referred to the web version of this paper.)

We now discuss the similarities and differences between the structure of acetyl-YEQGL-amide determined here and the previously reported structure of a 10-residue peptide containing the YEQGL sequence (VEDYEQGLSG; residues 375–384 of rat P2X4) complexed with the μ2 subunit of adaptor protein 2 (Royle et al., 2005). Aligning the two structures along the peptide backbone of glutamine and glycine (Fig. 4a) reveals that only the sidechains of glutamine are superimposed in the two structures, whereas the tyrosine, glutamic acid and leucine sidechains all adopt significantly different conformations. In the structure of acetyl-YEQGL-amide, the molecule adopts a more elongated conformation, whereas in the structure of the peptide-protein complex, the molecule curves around the μ2 subunit binding site (Royle et al., 2005) (Fig. 4c).

In the peptide–protein complex, the hydrogen-bonding arrangement comprises five significant interactions (Fig. 4b). The OH group of tyrosine is engaged in a hydrogen bonded triad with Asp^176^ and Lys^203^ of μ2, the peptide backbone of glutamic acid forms two hydrogen bonds with the backbone of Val^422^, the backbone of glycine forms a hydrogen bond with Lys^420^, and the backbone of leucine forms a hydrogen bond with a water molecule next to the peptide binding site. Visualizing these interactions on the surface of μ2 demonstrates the specificity of this protein for the YXXΦ or YXXGΦ motif (Fig. 4c). Tyrosine is recognized in a specific binding pocket by strong hydrogen bonding. The peptide backbone of two amino acids (or, in the case of YEQGL, two amino acids plus glycine) interacts with the surface of μ2, with the sidechains pointing away from the protein, permitting an amino-acid-independent interaction. Finally, leucine is held in a second specific binding pocket by hydrophobic interactions. As a result of these interactions, the YEQGL sequence is “wrapped” around the binding site of μ2.

Although the two structures differ in several aspects, there are nevertheless notable similarities. In particular, hydrophobic interactions involving the leucine sidechain (*via* intermolecular interactions with hydrophobic regions of neighbouring molecules in the case of acetyl-YEQGL-amide, or with a hydrophobic pocket in the case of the peptide–protein complex) are key structural elements for YEQGL. Essentially, the structure of acetyl-YEQGL-amide differs from the structure of the peptide-protein complex in the way in which the hydrogen bonding and hydrophobic interactions between the tyrosine and leucine sidechains are accommodated. In the peptide–protein complex, the tyrosine and leucine in the YEQGL region interact with specific binding pockets on the surface of the μ2 protein causing the sequence to wrap around the protein binding site, which probably changes the structure of this region significantly relative to the native conformation.

It is reasonable to consider whether the crystal structure of acetyl-YEQGL-amide bears any resemblance to the structure of the YEQGL region within the C-terminus of P2X4. At present, no high-resolution structural data are available for the C-termini of P2X receptors, so this issue cannot be assessed definitively. In the case of zebrafish P2X4.1, it was necessary to truncate the C-terminus in order to obtain crystals that diffract well (Kawate et al., 2009), suggesting that this region of the protein may be flexible under native conditions. However, the YEQGL sequence is not present in the C-terminus of zebrafish P2X4.1, and it is possible that the C-termini of mammalian and fish P2X4 receptors adopt different structures or engage in different interactions with other proteins *in vivo*. Nevertheless, it is certain that the structure of acetyl-YEQGL-amide determined here must represent an accessible, low-energy conformation for this molecule, which may represent the conformation of this region in the absence of interacting proteins. Finally, we note that CD measurements carried out here for acetyl-YEQGL-amide in solution (see Supplementary Fig. 1) suggest that this peptide has a preference for a random coil conformation in the solution state.

## 4 Concluding remarks

The crystal structure of acetyl-YEQGL-amide reported here provides a successful demonstration of the applicability of modern techniques for carrying out *ab initio* structure determination of biologically relevant oligopeptides directly from powder X-ray diffraction data. Nevertheless, it is important to note that the technical challenges associated with *ab initio* structure solution from powder X-ray diffraction data are such that, at present, this technique is most applicable in cases of relatively short peptide sequences. In the present work, we selected a short model peptide (acetyl-YEQGL-amide) for which the sequence has a known biological role and for which the structure of a complex with its interacting partner (the μ2 subunit of AP2) was already available (Royle et al., 2005) for comparison.

The next stage in the development of powder X-ray diffraction in structural biology will involve the extension of this methodology to study larger oligopeptides, representing more challenging structure determination problems, with the ultimate goal of achieving *ab initio* structure solution of small proteins in the future, thus circumventing the need to grow single crystals of sufficient size for conventional protein crystallography. In this regard, it is relevant to note that considerable progress has been made in recent years in several aspects of the study of proteins by powder X-ray diffraction (Margiolaki and Wright, 2008), including the optimization of conditions for recording high-quality powder X-ray diffraction data for such materials using synchrotron radiation sources. In favourable cases, the quality of data recorded has been shown to be adequate to allow successful indexing and structure refinement (the latter starting from a known structural model, including the application of “molecular replacement” strategies to define the starting model for structure refinement) (Basso et al., 2005, Margiolaki et al., 2005, Margiolaki et al., 2007, Von Dreele, 1999, Von Dreele, 2001, Von Dreele et al., 2006). Although complete *ab initio* structure solution of a protein structure from powder X-ray diffraction data has not yet been reported, continued progress in the development of methodology in this field (including direct-space structure solution methodology of the type employed in the present work) promises to pave the way for achieving this goal in the future.

## References

[b0005] Albesa-Jové D., Kariuki B.M., Kitchin S.J., Grice L., Cheung E.Y., Harris K.D.M. (2004). Challenges in direct-space structure determination from powder diffraction data: a molecular material with four independent molecules in the asymmetric unit. Chem. Phys. Chem..

[b0010] Altomare A., Caliandro R., Cuocci C., Giacovazzo C., Moliterni A.G.G., Rizzi R., Platteau C. (2008). Direct methods and simulated annealing: a hybrid approach for powder diffraction data. J. Appl. Crystallogr..

[b0015] Ban N., Nissen P., Hansen J., Moore P.B., Steitz T.A. (2000). The complete atomic structure of the large ribosomal subunit at 2.4 Å resolution. Science.

[b0020] Basso S., Fitch A.N., Fox G.C., Margiolaki I., Wright J.P. (2005). High-throughput phase-diagram mapping via powder diffraction: a case study of HEWL versus pH. Acta Crystallogr. D.

[b0025] Bobanovic L.K., Royle S.J., Murrell-Lagnado R.D. (2002). P2X receptor trafficking in neurons is subunit specific. J. Neurosci..

[b0030] Bonifacino J.S., Dell’Angelica E.C. (1999). Molecular bases for the recognition of tyrosine-based sorting signals. J. Cell. Biol..

[b0035] Boultif A., Louër D. (2004). Powder pattern indexing with the dichotomy method. J. Appl. Crystallogr..

[b0040] Brodski V., Peschar R., Schenk H. (2005). Organa – a program package for structure determination from powder diffraction data by direct-space methods. J. Appl. Crystallogr..

[b0045] Brunelli M., Wright J.P., Vaughan G.B., Mora A.J., Fitch A.N. (2003). Solving larger molecular crystal structures from powder diffraction data by exploiting anisotropic thermal expansion. Angew Chemie Int. Ed..

[b0050] Chernyshev V.V. (2001). Structure determination from powder diffraction. Russ. Chem. Bull..

[b0055] Cheung E.Y., Harris K.D.M., Kang T., Scheffer J.R., Trotter J. (2006). Structure-reactivity correlations for solid-state enantioselective photochemical reactions established directly from powder X-ray diffraction. J. Am. Chem. Soc..

[b0060] Cheung E.Y., McCabe E.E., Harris K.D.M., Johnston R.L., Tedesco E., Raja K.M.P., Balaram P. (2002). C−H…O hydrogen bond mediated chain reversal in a peptide containing a γ-amino acid residue, determined directly from powder X-ray diffraction data. Angew Chemie Int. Ed..

[b0065] Crowfoot Hodgkin D., Pickworth J., Robertson J.H., Trueblood K.N., Prosen R.J., White J.G. (1955). The crystal structure of the hexacarboxylic acid derived from B_12_ and the molecular structure of the vitamin. Nature.

[b0070] David W.I.F., Shankland K. (2008). Structure determination from powder diffraction data. Acta Crystallogr. A.

[b0075] David W.I.F., Shankland K., McCusker L.B., Baerlocher C. (2002).

[b0080] Deisenhofer J., Epp O., Miki K., Huber R., Michel H. (1984). X-ray structure analysis of a membrane protein complex: Electron density map at 3 Å resolution and a model of the chromophores of the photosynthetic reaction center from *Rhodopseudomonas viridis*. J. Mol. Biol..

[b0085] Denlinger L.C., Fisette P.L., Sommer J.A., Watters J.J., Prabhu U., Dubyak G.R., Proctor R.A., Bertics P.J. (2001). Cutting edge: the nucleotide receptor P2X7 contains multiple protein- and lipid-interaction motifs including a potential binding site for bacterial lipopolysaccharide. J. Immunol..

[b0090] Favre-Nicolin V., Černý R. (2004). A better FOX: using flexible modelling and maximum likelihood to improve direct-space ab initio structure determination from powder diffraction. Z. Kristallogr..

[b0095] Fujii K., Uekusa H., Itoda N., Hasegawa G., Yonemochi E., Terada K., Pan Z., Harris K.D.M. (2010). Physicochemical understanding of polymorphism and solid-state dehydration/rehydration processes for the pharmaceutical material acrinol, by ab initio powder X-ray diffraction analysis and other techniques. J. Phys. Chem. C.

[b0100] Guo F., Harris K.D.M. (2005). Structural understanding of a molecular material that is accessed only by a solid-state desolvation process: the scope of modern powder X-ray diffraction techniques. J. Am. Chem. Soc..

[b0105] Guo F., Martí-Rujas J., Pan Z., Hughes C.E., Harris K.D.M. (2008). Direct structural understanding of a topochemical solid state photopolymerization reaction. J. Phys. Chem. C.

[b0110] Habershon S., Harris K.D.M., Johnston R.L. (2003). Development of a multipopulation parallel genetic algorithm for structure solution from powder diffraction data. J. Comput. Chem..

[b0115] Hammond R.B., Roberts K.J., Docherty R., Edmondson M. (1997). Computationally assisted structure determination for molecular materials from X-ray powder diffraction data. J. Phys. Chem. B.

[b0120] Harris K.D.M. (2003). New opportunities for structure determination of molecular materials directly from powder diffraction data. Cryst. Growth Des..

[b0125] Harris K.D.M. (2009). Structure solution from powder X-ray diffraction data by genetic algorithm techniques, applied to organic materials generated as polycrystalline products from solid state processes. Mater. Manuf. Process.

[b0130] Harris K.D.M., Cheung E.Y. (2004). How to determine structures when single crystals cannot be grown: opportunities for structure determination of molecular materials using powder diffraction data. Chem. Soc. Rev..

[b0135] Harris K.D.M., Johnston R.L., Kariuki B.M. (1998). The genetic algorithm: foundations and applications in structure solution from powder diffraction data. Acta Crystallogr. A.

[b0140] Harris K.D.M., Tremayne M., Lightfoot P., Bruce P.G. (1994). Crystal Structure Determination from Powder Diffraction Data by Monte Carlo Methods. J. Am. Chem. Soc..

[b0145] Harris K.D.M., Habershon S., Cheung E.Y., Johnston R.L. (2004). Developments in genetic algorithm techniques for structure solution from powder diffraction data. Z. Kristallogr..

[b0150] Huq A., Stephens P.W. (2003). Subtleties in crystal structure solution from powder diffraction data using simulated annealing: ranitidine hydrochloride. J. Pharm. Sci..

[b0155] Kariuki B.M., Zin D.M.S., Tremayne M., Harris K.D.M. (1996). Crystal structure solution from powder X-ray diffraction data: the development of Monte Carlo methods to solve the crystal structure of the γ-phase of 3-chloro-trans-cinnamic acid. Chem. Mater..

[b0160] Kariuki B.M., Serrano-González H., Johnston R.L., Harris K.D.M. (1997). The application of a genetic algorithm for solving crystal structures from powder diffraction data. Chem. Phys. Lett..

[b0165] Kariuki B.M., Psallidas K., Harris K.D.M., Johnston R.L., Lancaster R.W., Staniforth S.E., Cooper S.M. (1999). Structure determination of a steroid directly from powder diffraction data. Chem. Commun..

[b0170] Kawate T., Michel J.C., Birdsong W.T., Gouaux E. (2009). Crystal structure of the ATP-gated P2X4 ion channel in the closed state. Nature.

[b0175] Kendrew J.C., Bodo G., Dintzis H.M., Parrish R.G., Wyckoff H., Phillips D.C. (1958). A three-dimensional model of the Myoglobin molecule obtained by X-ray analysis. Nature.

[b0180] Khakh B.S., North R.A. (2006). P2X receptors as cell-surface ATP sensors in health and disease. Nature.

[b0185] Larson A.C., Von Dreele R.B. (1994). General Structure Analysis System (GSAS). Los Alamos National Laboratory Report LAUR.

[b0190] Le Bail A., Duroy H., Fourquet J.L. (1988). Ab-initio structure determination of LiSbWO_6_ by X-ray powder diffraction. Mater. Res. Bull..

[b0195] Lightfoot P., Tremayne M., Harris K.D.M., Bruce P.G. (1992). Determination of a molecular crystal structure by X-ray powder diffraction on a conventional laboratory instrument. J. Chem. Soc. Chem. Commun..

[b0200] Margiolaki I., Wright J.P. (2008). Powder crystallography on macromolecules. Acta Crystallogr. A.

[b0205] Margiolaki I., Wright J.P., Fitch A.N., Fox G.C., Von Dreele R.B. (2005). Synchrotron X-ray powder diffraction study of hexagonal turkey egg-white lysozyme. Acta Crystallogr. D.

[b0210] Margiolaki I., Wright J.P., Wilmanns M., Fitch A.N., Pinotsis N. (2007). Second SH3 domain of ponsin solved from powder diffraction. J. Am. Chem. Soc..

[b0215] McCusker L.B., Von Dreele R.B., Cox D.E., Louër D., Scardi P. (1999). Rietveld refinement guidelines. J. Appl. Crystallogr..

[b0220] Pan Z., Xu M., Cheung E.Y., Harris K.D.M., Constable E.C., Housecroft C.E. (2006). Understanding the structural properties of a dendrimeric material directly from powder X-ray diffraction data. J. Phys. Chem. B.

[b0225] Perutz M.F., Rossmann M.G., Cullis A.F., Muirhead H., Will G., North A.C.T. (1960). Structure of haemoglobin: a three-dimensional Fourier synthesis at 55 Å resolution obtained by X-ray analysis. Nature.

[b0230] Rietveld H.M. (1969). A profile refinement method for nuclear and magnetic structures. J. Appl. Crystallogr..

[b0235] Royle S.J., Qureshi O.S., Bobanovic L.K., Evans P.R., Owen D.J., Murrell-Lagnado R.D. (2005). Non-canonical YXXGPhi endocytic motifs: recognition by AP2 and preferential utilization in P2X4 receptors. J. Cell. Sci..

[b0240] Schluenzen F., Tocilj A., Zarivach R., Harms J., Gluehmann M., Janell D., Bashan A., Bartels H., Agmon I., Franceschi F., Yonath A. (2000). Structure of functionally activated small ribosomal subunit at 3.3 Å resolution. Cell.

[b0245] Seebach D., Matthews J.L., Meden A., Wessels T., Baerlocher C., McCusker L.B. (1997). Cyclo-beta-peptides: structure and tubular stacking of cyclic tetramers of 3-aminobutanoic acid as determined from powder diffraction data. Helv. Chim. Acta.

[b0250] Surprenant A., North R.A. (2009). Signaling at purinergic P2X receptors. Ann. Rev. Physiol..

[b0255] Tedesco E., Turner G.W., Harris K.D.M., Johnston R.L., Kariuki B.M. (2000). Structure determination of an oligopeptide directly from powder diffraction data. Angew Chemie Int. Ed..

[b0260] Tedesco E., Harris K.D.M., Johnston R.L., Turner G.W., Raja K.M.P., Balaram P. (2001). Structure determination of a peptide beta-turn from powder X-ray diffraction data. Chem. Commun..

[b0265] Tedesco E., Sala F.D., Favaretto L., Barbarella G., Albesa-Jové D., Pisignano D., Gigli G., Cingolani R., Harris K.D.M. (2003). Solid-state supramolecular organization, established directly from powder diffraction data, and photoluminescence efficiency of rigid-core oligothiophene-S, S-dioxides. J. Am. Chem. Soc..

[b0270] Tremayne M. (2004). The impact of powder diffraction on the structural characterization of organic crystalline materials. Phil. Trans. R Soc. Lond..

[b0275] Tsue H., Horiguchi M., Tamura R., Fujii K., Uekusa H. (2007). Crystal structure solution of organic compounds from X-ray powder diffraction data. J. Synth. Org. Chem. Japan.

[b0280] Turner G.W., Tedesco E., Harris K.D.M., Johnston R.L., Kariuki B.M. (2000). Implementation of Lamarckian concepts in a Genetic Algorithm for structure solution from powder diffraction data. Chem. Phys. Lett..

[b0285] Von Dreele R.B. (1999). Combined Rietveld and stereochemical restraint refinement of a protein crystal structure. J. Appl. Crystallogr..

[b0290] Von Dreele R.B. (2001). Binding of N-acetylglucosamine to chicken egg lysozyme: a powder diffraction study. Acta Crystallogr. D.

[b0295] Von Dreele R.B., Lee P.L., Zhang Y. (2006). Protein polycrystallography. Z. Kristallogr..

[b0300] Wimberly B.T., Brodersen D.E., Clemons W.M., Morgan-Warren R.J., Carter A.P., Vonrheln C., Hartsch T., Ramakrishnan V. (2000). Structure of the 30S ribosomal subunit. Nature.

[b0305] Young R.A. (1993).

