# Supplementary material for: Exploiting powder X-ray diffraction for direct structure determination in structural biology: The P2X4 receptor trafficking motif YEQGL
Source: J Struct Biol. 2011 Jun;174(3):461–7. doi: 10.1016/j.jsb.2011.03.001 (PMC3121957; doi:10.1016/j.jsb.2011.03.001)

Caption for Supplementary Figure

Supplementary Figure 1 CD spectrum of acetyl-YEQGL-amide in 25 mM potassium phosphate buffer (pH 7.5). The spectrum shown is an average of 11 traces. There is no evidence for any significant amounts of either α- or β-structure, and the peptide appears to be predominantly in the random coil conformation.

Supplementary Figure 1

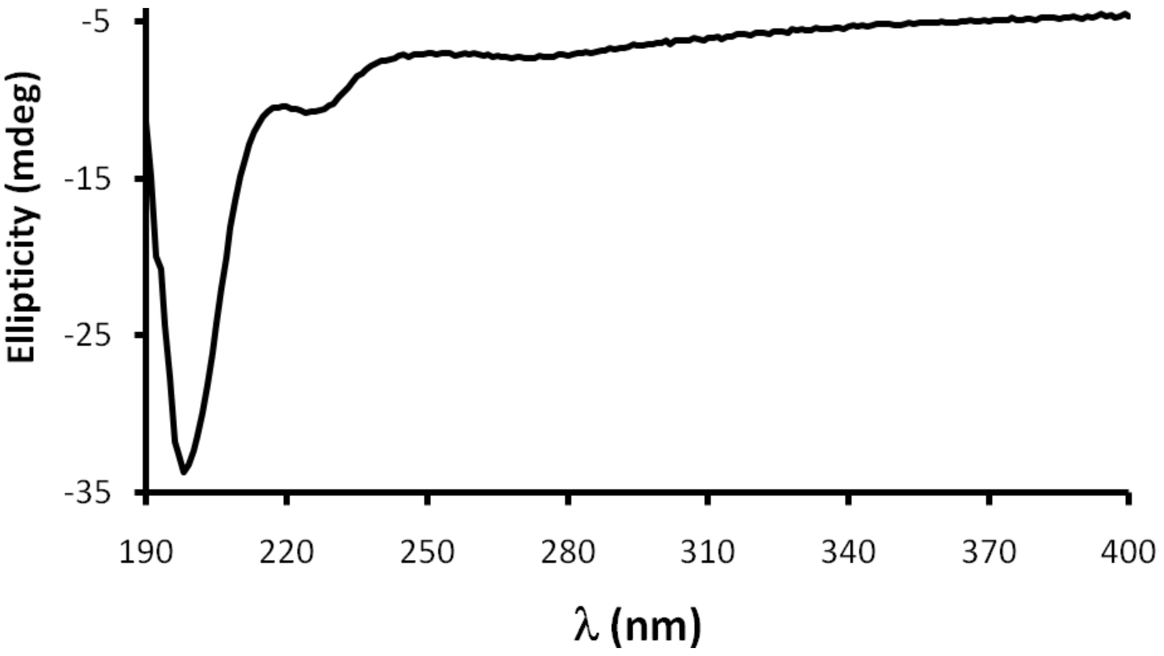

Supplement: Supplementary data 1 [file mmc1.pdf]
